# Antiplasmodial Effect of *Anthocleista vogelii* on Albino Mice Experimentally Infected with *Plasmodium berghei berghei* (NK 65)

**DOI:** 10.1155/2014/731906

**Published:** 2014-05-11

**Authors:** Lebari Barine Gboeloh, Okpok Eta Okon, Samuel Effiong Udoh

**Affiliations:** ^1^Department of Biology, Ignatius Ajuru University of Education, Port Harcourt, Rivers State, Nigeria; ^2^Department of Zoology and Environmental Biology, University of Calabar, Cross River State, Nigeria; ^3^Cross River University of Technology, Calabar, Cross River State, Nigeria

## Abstract

The objective of the present study was to investigate the antiplasmodial effect of the ethanolic stem bark extract of *Anthocleista vogelii* at different doses in albino mice infected with *Plasmodium berghei berghei* (NK 65). Thirty-six mice were divided into six groups of six mice each. Five groups (B_1_–B_3_, D, and G) were infected with *Plasmodium berghei berghei* parasitized red blood cells. Groups D, H, and G served as the controls. Six days after infection, mice in groups B_1_, B_2_, and B_3_ were treated orally with 100, 200, and 400 mg/kg body weight of *Anthocleista vogelii,* respectively, for six executive days. Group D was treated with 5 mg/kg body weight of chloroquine while Group G was given distilled water. Group H was not infected and was not treated. It served as the normal control. The extracts exhibited significant (*P* < 0.05) dose-dependent chemosuppression of *P. berghei*. The extract exhibited average chemosuppressive effects of 48.5%, 78.5%, and 86.6% at dose levels of 100, 200, and 400 mg/kg body weight, respectively. Phytochemical screening of the plant extract revealed the presence of saponins, cardiac glycosides, flavonoids, terpenes, alkaloids, and steroid. The acute toxicity (LD_50_) of the plant was estimated to be 3162 mg/kg body weight. It showed that the stem bark of *A. vogelii* possesses antiplasmodial property.

## 1. Introduction

Malaria, an infectious disease associated with fever, anaemia, and other pathologies, is caused by species of* Plasmodium*. This genus infects mammals, birds, and lizards and is transmitted by the bite of female mosquitoes (*Anopheles* species in mammals or* Culex* species in birds and lizards) in which part of the life cycle is spent. Worldwide, clinical cases of malaria were observed in about 270 million people annually resulting in at least 1.5–2.7 million deaths a year [1]. There were approximately 3.3 billion people at risk of developing malaria each year, with at least 500 million cases, and nearly a million deaths annually worldwide [2]. This averages one person dying of malaria every 30 seconds. Over 90% of deaths occur within the continent of Africa, mainly among young children [3]. Despite efforts to reduce transmission and increase treatment, there has been little change in areas at risk of this disease [4]. Precise statistics are unknown about increasing prevalence of malaria because many cases occur in rural areas where people do not have access to hospitals or the means to afford health care; hence, majority of cases are undocumented [5].

In Nigeria, the burden of malaria is well documented and has been shown to be a big contributor to the economic burden of disease in communities where it is endemic and is responsible for annual economic loss of 132 billion Naira [6, 7]. It is estimated that 300,000 deaths occur each year, and 60% of outpatient visits and 30% hospitalizations are all attributed to malaria [8]. About 50% of the population has at least one episode of malaria annually resulting in high productivity losses [8, 9]. The disease is particularly virulent among pregnant women and children under 5 years of age due to their low levels of immunity [1]. The trend is rapidly increasing due to the current malaria resistance to first line antimalarial drugs [10]. It is responsible for over 90% of reported cases of tropical disease in Nigeria [11, 12].

The efficacy of these conventional drugs against malaria parasite has been reported with variable successes [13, 14]. The toxic effects of these chemicals on humans [15, 16], the development of resistance to it by target parasites [17], and the high cost of drugs [18] have paved way for herbal remedies as reasonable alternative.

Numerous plants indigenous to Nigeria have been found with amazing antimalarial properties. It is therefore highly essential that indigenous plants used by the local people to treat malaria be scientifically investigated to prove their ethnotherapeutic use.

Many species of the genus* Anthocleista* are used in the treatment of malaria in South-South Nigeria.* Anthocleista vogelii* (family: Loganiaceae) is a tree, about 15–20 m in height, found mostly in the tropical rainforest. The stem bark decoctions are used traditionally in the treatment of malaria [19], as an antihelminthic especially for roundworms [20], as antidiarrhoea [21], and for treatment of epilepsy [22].

The present study seeks to evaluate the antiplasmodial activity of the ethanolic extract of the stem bark of* Anthocleista vogelii* at different dose levels in albino mice infected with* Plasmodium berghei berghei*.

## 2. Materials and Methods

### 2.1. Collection of Plant Materials

The stem bark of* A. vogelii* was obtained from Awi forest, Akamkpa Local Government Area of Cross River State, Nigeria. The plants were properly identified using appropriate identification keys. Voucher specimen of the plant was deposited in the herbarium of the Department of Botany, Faculty of Science, University of Calabar, Calabar, Nigeria.

### 2.2. Preparation of Powdered Stem Bark and Ethanolic Stem Bark Extract of* Anthocleista vogelii*

Fresh stem bark of* A. vogelii* was washed with clean water and air dried under shade for five days to reduce the water content. The dried stem bark was ground into powder in a mortar with pestle before being pulverized into powder form using an electric blender. About 300 g of the powdered sample was macerated in 80% ethanol in a plastic bottle. The mixture was allowed to stand overnight and the supernatant was carefully drained into a small cleaned stainless basin and evaporated to dryness in a water bath at 45°C overnight.

### 2.3. Phytochemical Analysis of the Plant Extracts

Phytochemical analysis of the ethanolic stem bark extract of* A. vogelii* was carried out using the procedure described by [23–25]. The phytochemical analysis examined the presence of the following chemical parameters in the plant extracts: tannins, saponins, flavonoids, cardiac glycosides, phenols, and alkaloids. About 0.5–2 g of powdered/aqueous extracts of *A. vogelii* was boiled or mixed with various reagents depending on the chemical parameter to be investigated using methods described by [23–25]. The change in coloration determined the presence or absence of a particular parameter investigated.

### 2.4. Acute Toxicity (LD_50_)

The median lethal dose (LD_50_) of the stem bark extract of* A. vogelii *that will kill 50% of the animals in a population was determinedorally using the method described by [19]. The mice were divided into five groups of four mice eachweighing between 18 g and 20 g. The mice were subjected to 24 hours fasting (with only water) before administration of extracts. The powdered stem bark extract was dissolved in 20% Tween-80 and administered in doses of  500, 1,000, 2,000, 3,000, and 4,000 mg/kg body weight orally. The sixth group served as the control and received only 20% Tween-80. The mice were then observed for toxicity and fatalities within 72 hours. The LD_50_ was calculated using the formula of [26]:

(1) $$\begin{array}{c} {\text{LD}}_{50}=\sqrt{ab}, \end{array}$$

where

 
*a* = least tolerable dose;
 
*b* = maximum tolerable dose.

### 2.5. Ethical Consideration

Application for approval for the use of animals for this study was made to the ethical committee of the Faculty of Basic Medical Sciences, University of Uyo, Akwa Ibom State, Nigeria. The committee granted outright approval for this study as the objectives were very crucial to the tropical African continent.

### 2.6. Acquisition of* Plasmodium berghei berghei* and Mice

Mice already parasitized with* Plasmodium berghei berghei* (NK 65) were bought from National Institute for Medical Research (NIMR), Lagos, and were maintained alive. The mice for the study were obtained from the Animal House of the Faculty of Basic Medical Sciences, University of Uyo, Uyo, Nigeria. The mice were housed in standard cages in the laboratory and stabilized for 7 days during which they were fed with standard livestock feed (Vital Feed Growers) obtained from Brand Cereals and Oil Mills Limited, Bukuru, Jos, Nigeria, and clean drinking water. The study was conducted in the animal house and in the Department of Biochemistry, University of Uyo, Uyo. The mice were handled in accordance with the guidelines for the care and use of laboratory animals by [27].

### 2.7. Inoculation of the Mice with the Parasites

The mice parasitized with* Plasmodium berghei berghei* (Nk 65) were sacrificed after six days, having been observed to have shown clinical symptoms of malaria recording a parasitaemia of 67.2%. The mice were anaesthetized in a glass jar containing cotton wool soaked in chloroform. Blood was collected from the sacrificed mice by cardiac puncture using sterile syringes and needles. The blood was diluted in normal saline in the ratio of 1 : 10 (1 mL of blood in 10 mL of normal saline). The parasitized erythrocyte in volume of 0.3 mL was used to infect each of the experimental mice intraperitoneally six days before treatment.

At the commencement of the experiment, 36 albino mice weighing between 13 and 23 g were divided into 6 groups of 6 mice each. These were labelled as Groups B_1_, B_2_, and B_3_ and control Groups D, G, and H.

Groups B_1_, B_2_, and B_3_ were treated for 6 consecutive days with 100, 200, and 400 mg doses of extract of* A. vogelii*/kg body weight orally and daily, respectively. Three control groups were used. Control Group D was inoculated with the parasite and treated with 5 mg chloroquine/kg weight orally and daily. The chloroquine was obtained from Sigma-Aldrich Company, St Louis M O, USA. Group G was infected with the parasite but was not treated with any extract. Group H was neither infected with the parasite nor treated with the extract. The extract was administered for 6 days and, on the first day after administration, the mice were sacrificed, and blood was collected from each mouse in all the groups by cardiac puncture using sterile syringes and needles. Fresh blood from the sacrificed mice was used to make thin and thick blood films for parasite count and determination of parasitaemia.

### 2.8. Determination of Parasitaemia

Six days after inoculation of parasite,blood was collected from the tail of each mouse in the various groups before administration of extracts. This was used to make thin and thick blood smears to determine the baseline parasitaemia.

Percentage of parasitaemia was determined by counting the number of parasitized erythrocytes out of 200 erythrocytes in random fields of the microscope. Percentage parasitaemia and average percentage parasitaemia were calculated according to the following formula adopted by [28]:

(2) $$\begin{array}{c} \text{PP}=\frac{\text{Total}\;\text{number}\;\text{of}\;\text{PRBC}}{\text{Total}\;\text{number}\;\text{of}\;\text{RBC}}\times 100, \end{array}$$

where PP = percentage parasitaemia, PRBC = parasitized red blood cells, and RBC = red blood cells.

Average percentage parasitaemia is

(3) $$\begin{array}{c} \text{APP}=\frac{\text{APPC}-\text{APPT}}{\text{APPC}}\times 100, \end{array}$$

where APP = average percentage parasitaemia, APPC = average percentage parasitaemia in the control, and APPT = average percentage parasitaemia in the test group.

### 2.9. Determination of Percentage Average Suppression

The percentage average chemosuppression (AS) was determined using the method of [29]. It was calculated by subtracting the average percentage parasitaemia in the test group (APT) from average percentage parasitaemia in control Group G (infected untreated group) (APC). The value obtained was expressed as a percentage of the average percentage parasitaemia in the control Group G:

(4) $$\begin{array}{c} \text{AS}=\frac{\text{APC}-\text{APT}}{\text{APC}}\times 100. \end{array}$$

### 2.10. Data Analysis

All tests were performed at statistical significance of *P* < 0.05 using SPSS version 18.0 software package and values were expressed as mean ± SEM (standard error of mean) and comparisons were made using one-way ANOVA.

## 3. Results

### 3.1. Phytochemical Composition of* Anthocleista vogelii*

Results of the preliminary phytochemical test carried out on the ethanolic stem bark extract of* A. vogelii* showed the presence of saponins, cardiac glycosides, flavonoids, terpenes, alkaloids, and steroid (Table 1).

### 3.2. Acute Toxicity (LD_50_) of* A. vogelii*

The acute toxicity study of* A. vogelii* showed behavioural signs of toxicity at doses above 2000 mg/kg body weight. The percentage mortality of the mice ranged between 75 and 100% at doses of 3000–6000 mg/kg body weight (Table 2). The LD_50_ of* A. vogelii* was 3162 mg/kg body weight (Table 2).

### 3.3. Antiplasmodial Activity of the Ethanolic Stem Extract of* A. vogelii*

The extracts showed significant dose-dependent (*P* < 0.05) antiplasmodial activity at the various concentrations (100, 200, and 400 mg/kg body weight) administered with average chemosuppression of 48.5%, 78.5%, and 86.6%, respectively (Table 3). The extract at 400 mg/kg body weight performed similarly well (86.6%) as the standard drug, chloroquine (5 mg/kg/day), which produced 100% chemosuppression (Table 3).

## 4. Discussions

Studies of the antiplasmodial effect of* A. vogelii* were carried out on albino mice experimentally infected with* P. berghei berghei*. The choice of these plants was based on previous reports of their antiplasmodial property [30, 31]. Again, the remarkable activity of quinine and other related drugs and the success of artemisinin stimulated the search for new plant derived antimalarial drugs [19]. However, reported cases of drug resistance to these drugs made the search and development of alternative antimalarial drugs inevitable [32].

Phytochemical analysis of the stem bark extract of* A. vogelii* showed the presence of alkaloids, cardiac glycosides, flavonoids, terpenes, steroid, and saponins. These phytochemicals were previously reported by [19, 33, 34]. These phytochemical compounds were also reported in another species,* A. djalonensis* [31]. The presence of similar phytochemicals was recorded in* A. grandiflora* [35]. These constituents have been found in other natural products which exhibited antimalarial activity [36]. Plants that contain many phytochemicals with biological activities including alkaloids and flavonoids could serve as sources of antimalarial drugs [37]. Therefore, the antiplasmodial activity of* A. vogelii* could be attributed to the presence of these phytochemical compounds [30, 31].

The ethanolic stem bark extract of* A. vogelii* was well tolerated by the mice up to the dose level of 2000 mg/kg body weight within 24–72 hours. However, physical signs of toxicity were noticed in mice administered with 3000 mg/kg body weight of the extract 24–72 hours after administration. The LD_50_ of ethanolic stem bark of* A. vogelii *was estimated to be 3,162 mg/kg body weight far above the highest administered dose level of 400 mg/kg body weight. This indicated that the mice were safe with the different doses of the ethanolic stem extracts administered to them. Similar result was recorded using the methanolic stem bark extract of another species of the plant,* A. grandiflora* [35]. Similar result was reported within 30 minutes to 1 hour after administration of the ethanolic leaf extract of another species of the plant,* A. djalonensis,* at the dose level of 5 g/kg [31]. The results implied that the ethanolic stem bark of* A. vogelii *was toxic at doses above 3000 mg/kg body weight causing toxic effects and eventual death of the animals [38, 39].

Parasitaemia in the infected mice was monitored in all the groups using thick and thin blood films made from the tail vein of the mice. The percentage average parasitaemia showed high level of infection in all the groups after five days of inoculation of* P. berghei*. This result is consistent with previous reports of high percentage parasitaemia in* P. berghei* infected mice after five days and death of infected mice after seven days of inoculation [40, 41]. The high level of parasitaemia is an important feature of* Plasmodium* infection which could result in severe anaemia.

The starting parasitaemia for the three replicates B_1_, B_2_, and B_3_ before administration of ethanolic stem bark extracts of* A. vogelii* was 10.34%, 5.5%, and 7.3% as indicated in Table 3. After administration of extract, the results obtained from the present study showed that the ethanolic stem bark extract of* A. vogelii* exhibited significant (*P* < 0.05) chemosuppressive effect against* Plasmodium berghei berghei *infection in mice. At the dose level of 100, 200, and 400 mg/kg body weight, the extracts showed remarkable average chemosuppressive effect of 48.5%, 78.5%, and 86.6%, respectively. This result suggested that the extract possesses some active phytochemical compounds that have direct effects on the parasites and the effect is dose-dependent [19].

The significant (*P* < 0.05) dose-dependent chemosuppressive effect of* A. vogelii* observed in the study is in agreement with previous reports by [19, 31].* P. berghei* parasite densities of 12,000 *μ*L and 19,520 *μ*L were recorded in mice before treatment, but, with the administration of ethanolic leaf extract of* A. vogelii* at the dose levels of 250 mg/kg and 100 mg/kg, parasite density reduced to 2,350 *μ*L and 10,000 *μ*L, respectively [19]. The chemosuppressive activity of 68.20% was recorded for* A. grandiflora* in mice at a dose level of 700 mg/kg/day [35]. These results are indication that plants of the genus* Anthocleista* possess phytochemical constituents that have antiparasitic property.

In this study, chloroquine, used as a positive control, was observed to significantly (*P* < 0.05) decrease the parasitaemia in the infected mice at higher rate (100%) than the stem bark extract of* A. vogelii*. The mechanism of action of these extracts of* Anthocleista* species was however said to be similar to that of chloroquine, a standard antimalarial drug which induced the destruction of the asexual forms of the* Plasmodium *parasite [42]. The 100% chemosuppressive effect of chloroquine recorded in the present study showed that it is still one of the drugs of choice against malaria parasite [43]. This does not however rule out the chloroquine resistance to the* Plasmodium* species of mammals. Moreover, the fact that some rural settlements in Africa still rely more on the use of medicinal plants for the treatment of malaria led to the identification of* A. vogelii* which proved very effective against *P. berghei berghei* at the dose level of 400 mg/kg recording 86.6% average chemosuppression.

## 5. Conclusion

The result obtained in this study showed that ethanolic extract of the stem bark of* A. vogelii* showed a dose-dependent antiplasmodial activity. It was most effective at the dose level of 400 mg/kg body weight. This plant can be recommended for use since it possessed a high chemosuppressive effect against the malaria and can be obtained at relatively no cost from the forest.

## Figures and Tables

**Table 1:** Phytochemical composition of the ethanolic stem bark extract of *A. vogelii*.

S/number	Phytochemicals	*A. vogelii *(stem bark)
1	Tannins	−
2	Phlobatannins	−
3	Saponins	++
4	Anthraquinones	−
5	Cardiac glycosides	++
6	Flavonoids	+
7	Deoxy sugar	−
8	Terpenes	++
9	Alkaloids	−
10	Steroid	+

++: moderately present; +: present in trace; −: absent.

**Table 2:** Acute toxicity (LD_50_) of *A. vogelii *after 72 hours.

Group	Number of mice	Dosage (mg/kg/bw)	% mortality
1	4	1000	0
2	4	2000	0
3	4	3000	75
4	4	5000	100
5	4	6000	100
6	4	—	0

LD_50_ of *A. vogelii* (*x*) = $\sqrt{ab}$,

where *a* = maximum dose with 0% mortality = 2000;

*b* = minimum dose with 100% mortality = 5000;

*x* = $\sqrt{2000\times 5000}$ = $\sqrt{10000000}$ = 3162 mg/kg body weight.

**Table 3:** Baseline parasitaemia and chemosuppression.

Group	Extract	Dosage (mg/kg/bw)	Average parasitaemia before administration of extracts (%)	Average suppression after administration of extracts (%)
B1	*A. vogelii *	100	10.34 ± 0.39	48.5
B2	*A. vogelii *	200	5.5 ± 0.80	78.5
B3	*A. vogelii *	400	7.3 ± 3.39	86.6
D	Chloroquine	5	9.1 ± 2.90	100
G	—	—	6.2 ± 0.4	—
H	—	—	—	—

Values are means ± SEM of *n* = 4 values significantly different (*P* < 0.05) from control (Group G).
